# Adaptive plasticity in the mouse mandible

**DOI:** 10.1186/1471-2148-14-85

**Published:** 2014-04-18

**Authors:** Philip SL Anderson, Sabrina Renaud, Emily J Rayfield

**Affiliations:** 1 School of Earth Sciences, University of Bristol, Bristol, UK; 2 Laboratoire de Biométrie et Biologie Evolutive, CNRS, Université Lyon 1, 69622 Villeurbanne, France; 3 Department of Biology, Duke University, Durham, NC, USA

**Keywords:** Biomechanics, Mice, Jaws, Adaptation, Geometric morphometrics, Modularity

## Abstract

**Background:**

Plasticity, i.e. non-heritable morphological variation, enables organisms to modify the shape of their skeletal tissues in response to varying environmental stimuli. Plastic variation may also allow individuals to survive in the face of new environmental conditions, enabling the evolution of heritable adaptive traits. However, it is uncertain whether such a plastic response of morphology constitutes an evolutionary adaption itself. Here we investigate whether shape differences due to plastic bone remodelling have functionally advantageous biomechanical consequences in mouse mandibles. Shape characteristics of mandibles from two groups of inbred laboratory mice fed either rodent pellets or ground pellets mixed with jelly were assessed using geometric morphometrics and mechanical advantage measurements of jaw adductor musculature.

**Results:**

Mandibles raised on diets with differing food consistency showed significant differences in shape, which in turn altered their biomechanical profile. Mice raised on a soft food diet show a reduction in mechanical advantage relative to mice of the same inbred strain raised on a typical hard food diet. Further, the soft food eaters showed lower levels of integration between jaw regions, particularly between the molar and angular region relative to hard food eaters.

**Conclusions:**

Bone remodelling in mouse mandibles allows for significant shifts in biomechanical ability. Food consistency significantly influences this process in an adaptive direction, as mice raised on hard food develop jaws better suited to handle hard foods. This remodelling also affects the organisation of the mandible, as mice raised on soft food appear to be released from developmental constraints showing less overall integration than those raised on hard foods, but with a shift of integration towards the most solicited regions of the mandible facing such a food, namely the incisors. Our results illustrate how environmentally driven plasticity can lead to adaptive functional changes that increase biomechanical efficiency of food processing in the face of an increased solicitation. In contrast, decreased demand in terms of food processing seems to release developmental interactions between jaw parts involved in mastication, and may generate new patterns of co-variation, possibly opening new directions to subsequent selection. Overall, our results emphasize that mandible shape and integration evolved as parts of a complex system including mechanical loading food resource utilization and possibly foraging behaviour.

## Background

Adaptation is a key feature in evolution, since it constitutes the ability of organisms to face changes in their environment. It usually occurs as the result of natural selection, hence by the evolutionary process of screening of the fittest phenotypes within the natural variation occurring in a population, leading to a progressive increase in favourable genotypes from one generation to the next. However, the role of non-heritable variation, produced by plasticity, in this process is being increasingly recognized [1-5]. For example, plastic variation may allow individuals to survive in new conditions, a prerequisite for the evolution of heritable adaptive traits [6,7]. The evolutionary role of plasticity has often been emphasized in systems involving polyphenism [8]. However, phenotypic plasticity can also encompass subtle trait variation, such as the phenotypic response of bone to biomechanical function [9]. In this context, the term ‘adaptation’ is also used as the observation that bone is responding in an active manner to its functional environment, especially muscle activity, and that this constitutes an ‘adaptation’ of the bone to its function [10]. It is however unclear how these two notions of ‘adaptation’ relate to each other, i.e. if the direct ‘adaptation’ of a bone to its functional environment constitutes an evolutionary ‘adaptation’, i.e. an increase in fitness, corresponding to an increase in functional efficiency.

The present study aims to investigate this issue by focusing on bone remodelling in the mouse mandible. Note that strictly speaking, bone remodelling involves alteration of bone that has already been fully-grown as opposed to bone modelling which describes the initial growth of the bone into its adult morphology. For simplification, ‘bone remodelling’ is considered here as mechanisms affecting the mandibular bone in the adulthood of the mice, namely after weaning, when most growth is achieved [11]. That remodelling significantly affects mandible shape has been demonstrated by several experiments, most involving feeding rats or mice food of different consistency, ending with mandibles of different morphology [12-16]. It is unclear, however, if the remodelling occurring in this context has an evolutionary adaptive value, i.e. mice fed on hard food display mandibles that are functionally more efficient for food processing than mice fed on soft food. It is further unclear how these changes may relate to changes in mandible shape. A modular response of the mandible, i.e. localized in some parts, may be expected because (1) The anterior alveolar region and the posterior ramus arise from different developmental origins [17], making them modules with less integrated variation between them than within them [18]; (2) different parts of the functional apparatus of the mandible, including various adductor muscles, jaw joints, and incisor and molar teeth, act on the bone in a localized manner, leading to the recognition of functional modules nested within the developmental ones. However, bone remodelling and muscle activity, acting between parts that interact to perform a single function, may be a factor promoting integration of the mandible beyond genetic and developmental modularity [19,20].

**Figure 1:**
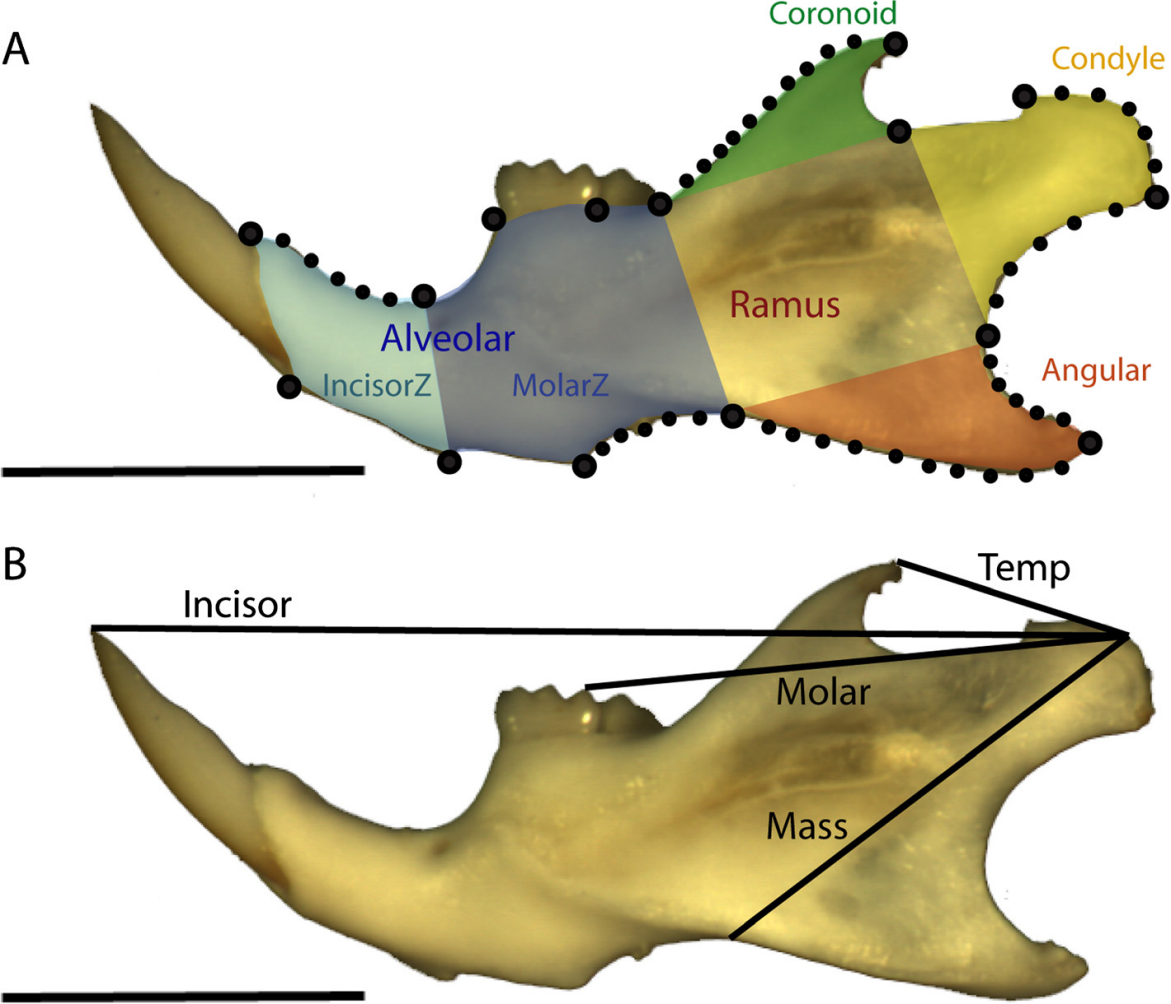
**Data collected for the morphometric and mechanical analyses the left mandible as an example. A**: Morphometric data based on 15 landmarks and 45 semi landmarks sampled over 7 curves. The shaded regions indicate the 5 hypothesized developmental and functional modules [13,25]. **B**: The two inlever lengths (based on the temporal and masseter muscle insertions) and the two outlever lengths (based on bite points at the incisors and molars). These are used in various combinations to measure four distinct mechanical advantages (Temporal-Incisor, Temporal-Molar, Masseter-Incisor, Masseter-Molar). All scale bars = 3 mm.

Using a sample of inbred laboratory mice fed on hard versus soft food [15], we performed a set of biomechanical and morphometric analyses (Figure 1). The hypotheses investigated were: (1) Mandibles of mice fed on diets of different consistency have different biomechanical trait values (four different measures of mechanical advantage; MA); and if bone remodelling has an adaptive value, then mandibles of mice fed on hard food (HF) should have higher mechanical advantage values than mice fed on soft food (SF); (2) Integration between parts of the mandibles should be higher in mice fed on hard food as this diet demands a higher level of masticatory function; (3) Parts directly involved in mastication (masseter muscles and molar zone) should show particularly strong integration.

## Methods

### Materials

Female mice from the inbred strain C56BL/6 J were ordered from the Charles River Laboratory. They were 3 weeks of age when obtained and were subsequently reared at the PBES (Ecole Normale Supérieure de Lyon, France) until the age of six months, after which they were sacrificed. At their arrival, the mice were randomly split into one group fed a standard hard pellet diet (hard food group, herein referred to as HF) and one group fed a diet consisting of ground pellets mixed with indigestible hydrated agar-agar jelly (soft food group, herein labeled SF) [15]. The resulting sample was 19 HF and 20 SF. For these mice, left mandibles were pictured when placed flat on the lingual side. Note that a mouse mandible is basically built on a single plane. A 2D quantification offers a very good approximation of its shape and biomechanical properties. The lingual side was chosen as it makes the muscle attachments visible for the biomechanical measurements. These pictures served for both, biomechanical and morphometric analyses.

The mice were provided with food and water *ad libidum* during the experiment. This protocol was evaluated by the internal committee of the PBES 'Validation de produit et protocole' (‘validation of products and protocols’). It stated that according to the European directive 2010/63/UE, such a protocol involving no pain inflicted to the mice did not require any formal ethical agreement. Mice were sacrificed according to the 2010/63/UE directive. Breeding conditions and hence animal welfare in the PBES was validated by a ministerial agreement (agreement B 69 123 0303 - 17/02/2009 of the French Ministère de l’Agriculture).

### Biomechanical data collection and statistics

We measured mechanical advantage, a measure of the efficiency of a jaw to transmit force from the muscle to the bite point [21-23]. Mechanical advantage is the ratio of the inlever (distance from the jaw joint to the point of muscle attachment) and the outlever (distance from the jaw joint to the bite point) and has long been used as a metric for mammalian jaw function [24,25]. There are two major muscle groups that operate to close the lower jaw in rodents: the masseter complex, attaching along the ramus and angular process, and the temporalis complex attaching along the coronoid process (Figure 1) [26]. Mice, like all rodents, also have two distinct dental regions. Anterior incisors are generally used for pre-oral processing as well as various non-feeding behaviours and the more posterior molar complex is used for chewing. In order to examine the biomechanical consequences of different diets we measured four distinct mechanical advantages on each jaw (Figure 1b): MA_T/I_ (inlever: temporalis insertion, outlever: incisor tip); MA_M/I_ (inlever: masseter insertion, outlever: incisor tip); MA_T/M_ (inlever: temporalis insertion, outlever: tip of the first molar hypoconid as the bite point); and MA_M/M_ (inlever: masseter, outlever: first molar hypoconid bite point). The tip of the coronoid process was used as a marker for the temporalis inlever and the anterior edge of the angular process (landmark 12) for the masseter inlever.

To test for significant differences in mechanical advantage values between experimental groups, we performed Student's t-tests and Kruskal-Wallis tests, the latter of which does not assume normal distributions. As mechanical advantage is a ratio, it is Cauchy distributed: the mean is undefined and it has an infinite variance [27]. Therefore, we regressed the numerator on to the denominator for each MA metric across the data set. Differences in the residuals from each regression were assessed by t-tests. This method tested the differences in the inlever of a given MA metric when the outlever is held at a constant value.

### Morphometric data collection and statistics

Outline morphometric analyses [15] previously demonstrated the absence of any significant size differences but found an overall shape difference between mandibles of mice fed on hard vs. soft food. In order to identify the local impact of remodelling on different parts of the mandible, as well as the possible role of biomechanical function on its integration, a further analysis was performed here based on a set of landmarks and semi- landmarks (Figure 1). Fifteen landmarks and 45 semi-landmarks defining seven curves were used to describe the shape of the mandible [19,20]. This data set can be split into two nested subsets corresponding to developmental and functional modules of the mandibles. The mandible is composed of two main modules, corresponding to the anterior, tooth-bearing part (alveolar region) and the posterior ramus [17,28]. It can be further split into five functional modules: the incisor and molar zone within the alveolar region, and the coronoid, condylar and angular processes composing the ramus [29] (Figure 1).

Landmarks and semi-landmarks were digitized using tpsDig 2.0 [30] and Procrustes superimposition was performed using tpsRelw [31]. Procrustes methods superimpose the landmarks in order to minimize differences between individuals based on position, rotation and scale. Semi-landmarks are slid along the curve until they are in positions that most closely match the reference configuration [32,33] based on minimizing the Procrustes distance [34-36]. This procedure produces new aligned coordinates for both the landmarks and semi-landmarks that can be used for further shape analyses.

The mandible was analysed as a whole, as alveolar + ramus region, and as five modules (incisor zone + molar zone + coronoid + condylar + angular processes). For each data-set, a separate Procrustes superimposition was performed. Principal component analyses (PCA) were used to summarize the total information on relevant axes representing more than 5% of the total variance. On this set of axes, the difference in shape between HF and SF mice was tested using multivariate analyses of variance (MANOVA) and a non-parametric equivalent (npMANOVA) testing the observed shape difference to differences between groups based on random permutations.

The strength of association between modules was evaluated using RV coefficients in HF and SF mice separately. The RV coefficient corresponds to the sum of the squared covariances between two sets of variables, divided by the total amount of variation in the two sets of variables [37]. The significance of the association was tested by comparing the observed RV coefficient to 9999 permutations. Statistics were performed using Systat, Past and ade4 [38].

## Results

### Biomechanical significance of the shape differences between mice fed hard vs. soft food

**Figure 2:**
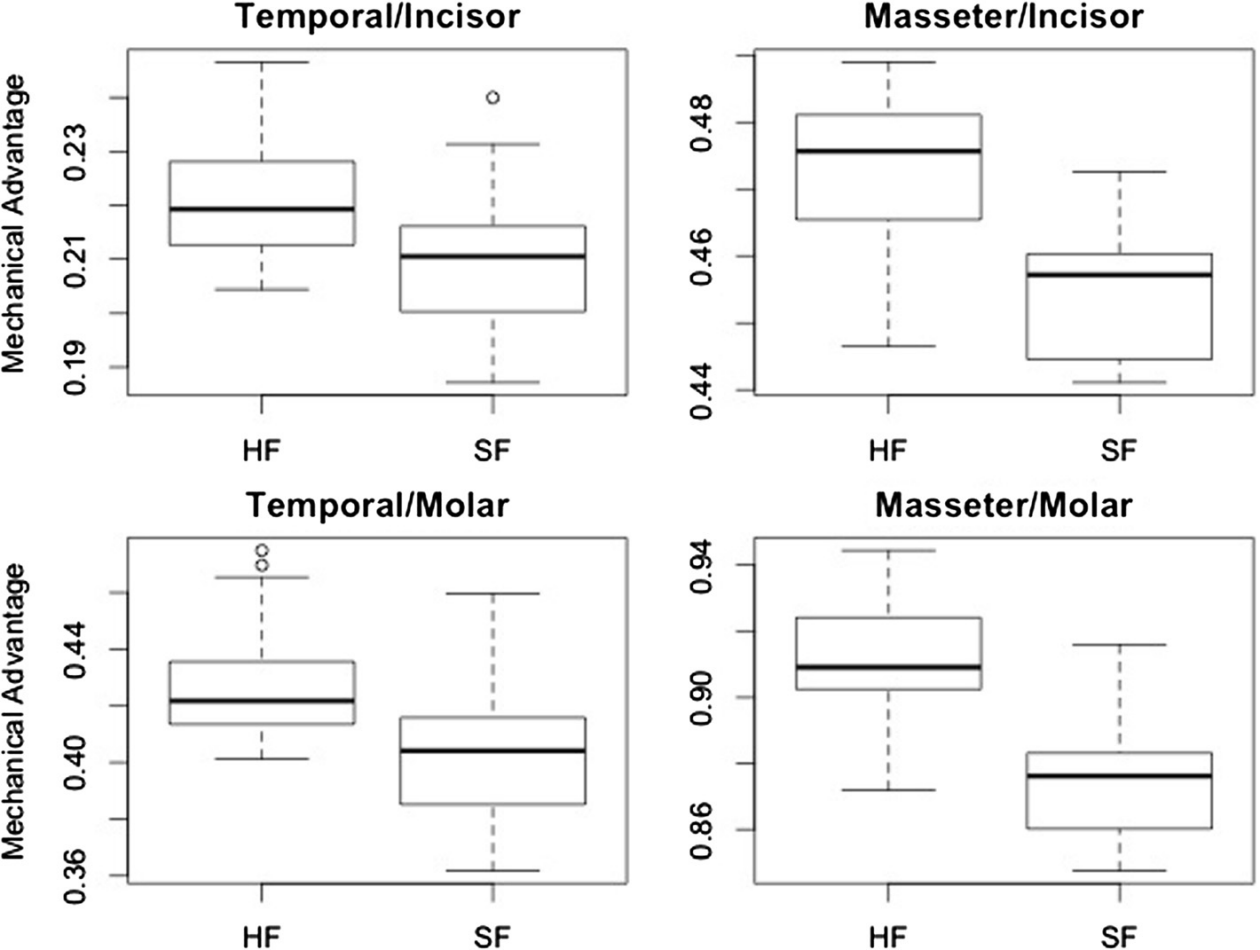
**Results of the mechanical analysis of mandible of mice reared on diets of different consistencies.** The animals fed a hard food diet show higher residual values (equivalent here to higher mechanical advantages) than the mice fed on soft food. This holds for all four mechanical advantage measures, although the difference is more pronounced when the masseter is used.

Between the dietary groups, the hard food eaters have significantly higher mechanical advantage (MA) values for all four MA measures (based on both the raw ratios and residuals from the regression of numerators on the denominators of these ratios, using both the Student’s t-test and Kruskal-Wallis tests: p < 0.01) (Figure 2). The difference is more pronounced in the MA measures which use the masseter muscle as the inforce (Figure 2).

### Shape differences between mice fed hard vs. soft food

**Figure 3:**
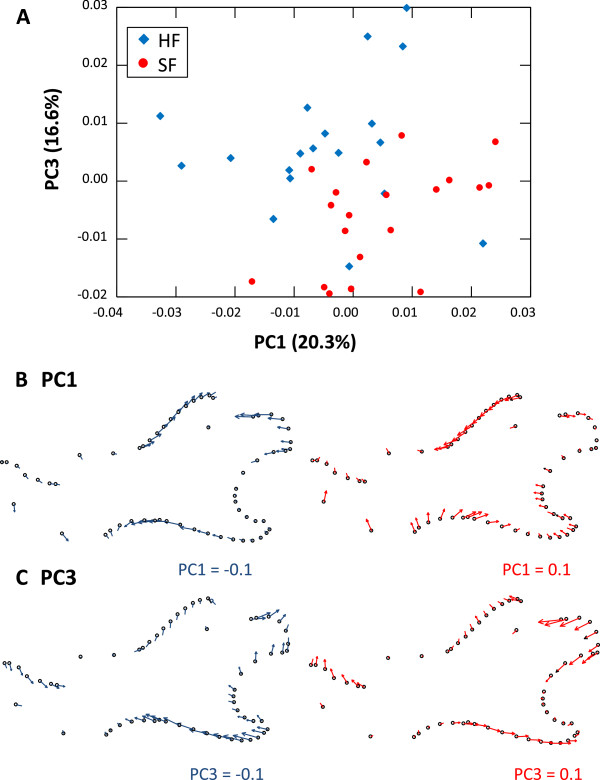
**Landmark-based analysis of the mandible shape of hard vs. soft food eaters. A**. Morphological variation in the space of the first and third principal components of the mandible shape analysis. Hard food eaters: blue diamonds; soft food eaters: red circles. **B** and **C**. Visualization of the deformation associated with the two axes. Deformation has been magnified by taking extreme values along the axes: −0.1 (in blue)/+0.1 (in red). **B**. Deformation along PC1. **C**. Deformation along PC3.

Based on principal component analyses on the residuals after Procrustes superimposition, between 3 and 6 axes were sufficient to summarize more than 95% of the total morphological variance (Table 1). As previously found using outline analyses, mandibles of mice fed hard vs. soft food were different in overall shape (Table 2; Figure 3). Both the alveolar region and the ramus were affected by this difference related to food consistency. Hard food eaters tend to display extended coronoid and angular processes, whereas the incisor and molar zones are expanded ventrally (Figure 3). All functional modules except the molar zone showed shape differences between HF and SF mice. The angular process displayed a reduced difference compared to other modules, possibly as it slightly changes its orientation compared to neighbouring modules without major changes to its own shape.

**Table 1:** Percentage of variance explained by principal components based on shape coordinates after Procrustes superimposition

**%var**	**RW1**	**RW2**	**RW3**	**RW4**	**RW5**	**RW6**	**RW7**
Mandib	20.4	17.2	15.1	8.8	6.4	5.1	3.5
Alveolar	40.3	15.2	11.1	6.1	4.6		
Ramus	24.0	19.6	14.0	11.1	6.6	4.5	
IncisorZ	48.6	16.0	13.4	7.3	6.1	3.2	
MolarZ	66.1	14.2	6.3	3.9			
Coronoid	66.0	15.3	7.2	4.5			
Condyle	38.9	21.8	9.3	8.3	7.3	3.8	
Angular	44.1	24.3	8.7	6.9	4.0		

Axes representing more than 5% of variance were considered in subsequent analyses.

**Table 2:** Differences in shape between hard and soft food eaters

	**Shape**	
	P_MANOVA_	PnpMANOVA
Mandib	**< 0.001**	**< 0.001**
Alveolar	**0.008**	**0.025**
Ramus	**< 0.001**	**< 0.001**
IncisorZ	**0.001**	**0.002**
MolarZ	0.316	0.160
Coronoid	**< 0.001**	0.113
Condyle	**< 0.001**	**<0.001**
Angular	**0.013**	**0.037**

Shape differences were tested on the set of PCs explaining more than 5% of the total variance, using a multivariate analysis of variance (MANOVA) and its non-parametric analogue (npMANOVA) based on 10000 permutations between Euclidean distances. Probabilities are provided; in bold those significant.

### Patterns of integration in mice fed hard vs. soft food

Regarding the partition of the mandible in two main modules (alveolar region + ramus), hard food eaters appeared to display a higher level of integration than soft food eaters (HF: RV = 0.577; p < 0.001; SF: RV = 0.315; p = 0.024). When partitioning the mandible into five functional modules a different pattern of integration emerged for HF and SF mice (Table 3). Hard food eaters displayed a significant integration between the molar zone and the angular process. Soft food eaters only displayed a significant, although weak integration between the incisor zone and the condyle.

**Table 3:** Integration between modules of the mandible for mice fed hard food (HF – upper panel) and soft food (SF – lower panel)

**HF**	**IncisorZ**	**MolarZ**	**Coronoid**	**Condyle**	**Angular**
IncisorZ	-	0.407	0.622	0.841	0.724
MolarZ	0.102	-	0.213	0.458	**< 0.001**
Coronoid	0.064	0.117	-	0.179	0.410
Condyle	0.072	0.097	0.150	-	0.106
Angular	0.088	**0.456**	0.101	0.223	-
**SF**	IncisorZ	MolarZ	Coronoid	Condyle	Angular
IncisorZ	-	0.221	0.302	**0.042**	0.679
MolarZ	0.163	-	0.338	0.561	0.116
Coronoid	0.155	0.100	-	0.664	0.599
Condyle	**0.301**	0.108	0.105	-	0.989
Angular	0.120	0.187	0.087	0.061	-

The strength of the association was evaluated using RV coefficient. Observed RV values are provided below the diagonal. Probabilities of significance were obtained by comparing observed values to separate permutations of individuals on each of the modules (9999 permutations, P above the diagonal). In bold, significant correlations at 5%.

## Discussion

### Biomechanical consequences of food consistency on bone remodelling and mandible shape

The present study demonstrates that differences in mandible shape generated by remodelling in the context of different food consistency generate a significant difference in biomechanical trait values. Moreover, these trait differences are of benefit to the hard food eaters, which display a higher mechanical advantage and therefore more efficient conversion of adductor muscle force to bite force than soft food eaters. This is in agreement with an adaptive value of remodelling, since the hard food diet is more mechanically resistant, and therefore requires higher relative bite forces to break down and process, which in turn should generate greater stresses (force per unit area) in the jaw. Indeed, the alteration of morphology in mice fed on an artificial soft food diet resulted in a decrease in the effective mechanical advantage as the efficiency of the jaw system to transfer force from the muscle to the bite point becomes less important.

How bone plasticity can account for this mechanical change is still unclear given our current understanding of bone remodelling. Other experiments on mice and rats demonstrated that a soft diet indeed resulted in a decreased development of feeding musculature [39]. The consequent reduction of the forces applied to the mandible has been shown previously to cause a reduction of bone mineral density [14] as well. The effect of this reduction in density could enhance the reduced biomechanical advantage of the jaw shape. The effect on degree of mineralization may be more complex, since enhanced forces stimulates bone growth and remodelling [12,39] and hence may increase the degree of mineralization, at least in young animals [10].

The shift in morphology indicates a shallowing and relative elongation of the SF jaws relative to the HF jaws (Figure 3). Shallowing will decrease the distance between the jaw articulation and the muscle insertions on the coronoid and angular processes (the inlevers) of the SF jaws, whilst lengthening increases the distance from the articulation to the dentition (the outlevers), hence reducing the mechanical advantage overall. A larger difference is seen in the mechanical advantage measures where the masseter insertion is used for the inlever, probably because the masseter inlever will be shortened most as the jaw becomes less deep (Figure 1b, 3). These morphological shifts in the SF jaws naturally decrease mechanical advantage as a consequence of the overall change in shape. Whatever the complexity of the processes involved, this underlines that the functional environment that a mandible develops under cannot be separated from the shape change observed.

### Mandible, muscles, feeding behaviour: a co-adapted system?

In their usual commensal habitat, mice typically feed mainly on seeds, flowers and vegetative parts of various plants, especially grasses, making them well-known pests for cereal harvests [40,41]. Rodent pellets have a consistency close to these natural foodstuffs. In contrast, the experiment introduced a set of mice to an unusual food consistency by feeding them on jelly.

As the soft food eaters display mandible shapes with lower biomechanical trait values than mice fed on normal consistency food, plasticity could therefore be seen as maladaptive in a situation departing too strongly from the “ancestral” diet. This may point to a role of plasticity in ensuring an efficient mandible shape that evolved in the context of a given range of food resources and associated muscular functioning. This underlines that mandible shape evolved under an average functional regime and that in that regime, the complex of jaw bone, masticatory muscles, teeth, and even behaviour, evolved as a co-adapted system. Bone remodelling would thus contribute to integrate the different components of this complex system. This is evidenced here by the higher integration observed in the mandible of mice fed their regular diet, when compared to those fed on the unusual soft food.

### Diet-related changes in integration: a facilitation of new patterns of selection?

In mice fed on hard food, the dominant pattern of co-variation related the molar zone with the angular process, where the masseter muscles insert, a pattern repeatedly found in mice [20,29]. These two areas are heavily involved in chewing, mobilized to process hard and/or resistant food. This functional requirement was relaxed in the mice fed on jelly, but demands related to the other functions of the mandible, namely occlusion with the incisors for gnawing remained.

In SF mice, the pattern of integration switched in favour of the incisor zone and condyle. Plasticity may thus change the pattern of integration among modules in an adaptively favourable way when facing new functional demands, while the reduction in mechanical advantage seen in the soft food eaters appears to be maladaptive. As such, plasticity may allow a new pattern of response to selection by releasing previous constraints and generating new associations [42]. This may have eventually led to the unusual jaw morphology observed in the few murine rodents that evolved towards the preponderant use of the incisor-related function [43].

### Evolutionary consequences of plasticity

The study demonstrated that the morphological response of the mandible to a change in function was indeed advantageous, in the sense that a diet harder to process triggered a response for a jaw with a more efficient biomechanical function. This is evidence of a potential adaptive role of plastic remodelling in the jaw. Mice are known to be highly adaptable animals that have colonized environments as extreme as sub-Antarctic islands [44,45]. This successful persistence in such environments probably involved behavioural plasticity, allowing them to establish feral populations instead of relying on their usual commensal way-of-life. This involves a component of plasticity in diet, and mice have been shown to switch their diet in some insular environments in order to adapt to the local resources [46-48]. Our results suggest that this plasticity in diet could induce a morphological response by bone remodelling, itself with an adaptive value enhancing the potential success of these mice facing new conditions. Behaviour is indeed considered a highly-plastic trait, allowing populations to persist in new environments in the first place, thus allowing evolution in non-plastic traits to take place [1]. In that context, morphology is typically considered as a non-plastic trait [1,3]. However, our results show that mice display a significant plasticity that could support plasticity in diet by an improved functional efficiency.

Jaw bone remodelling could thus be of evolutionary significance in allowing populations to better face the challenges of a new environment. Such a process has been suggested for small, possibly transient populations of mice on small islands where they face unusual food resources and display a mandibular morphology paralleling the plastic response to food consistency [49]. It could contribute to the fast response in mandible shape of insular mice tracking habitat changes on a sub-Antarctic island over a time-scale as short as two decades [50].

How plasticity resulting from bone remodelling is related to long-term evolution is however unclear. The plasticity-related shape changes observed here are different from directions of evolution observed on large islands with sustainable mouse populations over centuries up to millennia, suggesting that long-term evolution did not follow the direction of plasticity [49]. By allowing mice to successfully deal with a broad range of resources, plasticity may even preclude further morphological evolution in many cases [3]. Other traits such as bone density may also evolve to insure mandibular efficiency in a new evolutionary context, involving interplay between behaviour, muscles, teeth and bones. Understanding the relationship between short-term adaptive plasticity and long-term evolution requires further integrative studies bringing together evolutionary biology, functional morphology and ecology.

## Conclusions

Our results support the hypothesis that bone remodelling in mouse mandibles allows for significant shifts in biomechanical ability. Food consistency influences this morphological shift in an adaptive direction: mice raised on hard food develop jaws with higher mechanical advantage that are better suited to processing hard foods. Bone remodelling triggered by diet consistency also affects the organisation of the mandible. Mice raised on soft food show less overall integration implying that this diet released the jaws from a certain level of developmental constraint. These results illustrate how environmentally driven plasticity can lead to adaptive biomechanical changes that allow the animal to overcome environmental challenges: eating hard food causes the jaws to become better at processing hard foods. At the same time, decreased demand in terms of food processing appears to release developmental interactions between jaw parts potentially generating new patterns of co-variation and opening new avenues to selection.

## Competing interests

The authors declare that they have no competing interests.

## Authors’ contributions

PSLA conceived of the study, carried out the biomechanical and statistical analyses and drafted the manuscript. SR helped design the study, performed morphometric and modularity analyses and helped draft the manuscript. EJR participated in the design of the study and helped interpret the data. All authors edited and approved the final manuscript.
